# Investigating the Efficacy of a Community Support Network Rehabilitation Intervention for Improving Resiliency, Quality of Life, and Neurocognitive Function in Survivors of Intimate Partner Violence–Caused Brain Injury: Protocol for a Feasibility Study

**DOI:** 10.2196/54605

**Published:** 2024-05-24

**Authors:** Shambhu Prasad Adhikari, Tori N Stranges, Setareh Nouri Zadeh Tehrani, Shaun Porter, Karen Mason, Paul van Donkelaar

**Affiliations:** 1 School of Health and Exercise Sciences, University of British Columbia Kelowna, BC Canada; 2 ABI Wellness Langley, BC Canada; 3 Supporting Survivors of Abuse and Brain Injury Through Research (SOAR) Kelowna, BC Canada

**Keywords:** brain injury, cognitive functions, community support network, intimate partner violence, quality of life, rehabilitation

## Abstract

**Background:**

Globally, approximately 1 in 3 women experience intimate partner violence (IPV) in their lifetime. Brain injury (BI) is a common, yet often unrecognized, consequence of IPV. BIs caused by IPV tend to be mild, occur repetitively over the course of months or years, are remote in time, and result in chronic symptoms. Similar to BI from other causes, therapeutic treatment for women with IPV-caused BI (IPV-BI) is crucial to help resolve any physical or cognitive impairments, enhance the quality of life (QoL), and minimize longer-term neurodegeneration.

**Objective:**

This study aims to investigate the feasibility and efficacy of a community support network (CSN) rehabilitation intervention regarding its impact on resiliency, QoL, and neurocognitive function.

**Methods:**

In this pre- and postexperimental design, women (aged 18 to 50 years) who are survivors of IPV and IPV-BI will be recruited from various community organizations serving survivors of IPV. Exclusion criteria will include current pregnancy and any diagnosed neurological disorder known to affect cerebrovascular, neurocognitive, or sensorimotor function. A CSN rehabilitation intervention that includes aerobic exercise, cognitive training, mindfulness meditation, and counseling will be administered. A trauma-informed approach will be integrated into the design and implementation of the program. Furthermore, the program will include a participant navigator who will provide trauma- and violence-informed advocacy and systems navigation support to participants, in addition to facilitating a monthly peer support group. The intervention will be provided for 2.5 hours a day and 2 days a week for 3 months. Participants will complete psychological assessments and provide clinic-demographic information in the first assessment. In the second (before intervention), third (after intervention), and fourth (at follow-up) sessions, they will complete tests of resiliency, QoL, and neurocognition. The estimated sample size is 100. The objective of this study will be accomplished by quantitatively measuring resiliency, QoL, and neurocognition before and immediately after the intervention. A follow-up assessment will occur 3 months after the completion of the intervention to evaluate the maintenance of any improvements in function. One-way ANOVAs will be used to evaluate the intervention outcome across the testing times. Relationships among various variables will be explored using regression analysis.

**Results:**

We anticipate that the CSN rehabilitation intervention will be effective in improving resiliency, QoL, and neurocognitive function in women who have experienced IPV-BI. Furthermore, we anticipate that this intervention will be feasible in terms of study recruitment, adherence, and retention.

**Conclusions:**

The CSN rehabilitation intervention will have a positive impact on resiliency, QoL, and neurocognitive functions in survivors of IPV-BI. Subsequently, a comparative study will be conducted by recruiting a control group receiving usual care.

**International Registered Report Identifier (IRRID):**

PRR1-10.2196/54605

## Introduction

### Background

Intimate partner violence (IPV) is remarkably prevalent (for women, lifetime prevalence has been reported as high as 1 in 3, and 12-month prevalence has been reported as high as 1 in 10) [1], leading to a variety of negative physical and mental health outcomes, including posttraumatic stress disorder (PTSD), anxiety, and depression [2]. Brain injury (BI) resulting from violent blows to the head, face, and neck or episodes of strangulation is increasingly recognized as part of the IPV experience [3-5]. The prevalence of IPV-caused BI (IPV-BI) is estimated at >70% [6,7]. Furthermore, strangulation is a common mechanism of injury in IPV [8] and often indicates an increase in the severity of BI. As such, nonfatal strangulation (NFS) is an important risk factor for negative health outcomes in people who experience IPV [9]. NFS is commonly a repetitive rather than an isolated event, and is used to exert control by inducing feelings of pain and helplessness [10]. NFS can lead to BI by one or more of the following mechanisms: (1) *anoxic or hypoxic* BI due to compression of the trachea; (2) *ischemic* BI due to occlusion of arterial blood flow; (3) *hypoxic-ischemic* BI due to the combined compression of the trachea and the carotid arteries or due to the triggering of carotid sinus reflex leading to dysrhythmia or cardiac arrest, which subsequently could cause BI; and (4) *pressure-related BI* due to the occlusion of the jugular veins leading to venous congestion, increased intracranial pressure, blocking of the removal of metabolic byproducts, and pinpoint hemorrhage [7,11,12].

Survivors of IPV-BI demonstrate cognitive impairments (eg, deficits in memory, attention, reasoning, planning, and executive functioning) [13], psychological or emotional disturbances (eg, depression, anxiety, fatigue, and PTSD) [5,13], and sensorimotor problems (eg, paralysis or paresis of facial or extremity muscles, numbness, loss of sensation, muscle spasms, facial droop, and unilateral weakness) [14], which ultimately impacts the quality of life (QoL). Therefore, therapeutic treatment for survivors of IPV-BI is crucial, and any physical or cognitive impairment in these survivors requires therapeutic interventions similar to those for BIs from other injury mechanisms (eg, sport-related concussion and car accidents). Effective therapeutic interventions would not only improve the QoL of survivors of IPV-BI but also have the potential to prevent longer-term neurodegeneration if early detection is possible and timely treatment is provided [15].

In the case of BI from other causes, individuals often access rehabilitative resources through the health care system [16]. Given the goals of care outlined by the Canadian Medical Association, resources are positioned to assess and treat both short- and long-term effects of BI, with the overarching goal of complete recovery. Rehabilitation is individualized and may include a range of services spanning emergency care to physical or occupational therapy [17]; however, differential access is common. Women who have experienced IPV-BI are chronically underserved [4,7,18]. This is in part due to the failed recognition of IPV and an inability to link IPV and BI within the more comprehension health care system. The complexities surrounding IPV, such as safety, stigma, and child custody concerns, discourage survivors from accessing primary health care. If survivors do access primary health care, they may not feel comfortable or see marked benefit in disclosing the cause of their injuries [19]. Disclosure of IPV in health care settings is further influenced by negative health care provider attitudes, survivors’ perception of safety, and concerns about the consequences of disclosing. Importantly, help-seeking behaviors are intersectional and may also be influenced by race, ethnicity, gender identity, or sexual orientation [20]. As a result of these factors, evidence-based therapeutic interventions to improve the QoL of survivors of IPV-BI are still lacking. Therefore, in collaboration with ABI Wellness, our team has developed a rehabilitation program termed the “community support network” (CSN) rehabilitation intervention specific to IPV-BI. Traditionally, health care and community supports that offer services for those who have experienced IPV typically do not take IPV-BI into account. Recent Canadian research by Nicol et al [21] found that 75% of the women’s shelter workers in a national sample reported they did not screen for BI following IPV [21]. The challenge in assessing IPV-BI is further complicated by the increased risk of psychopathological comorbidities such as PTSD, depression, anxiety, and substance use that can mask or exacerbate BI symptoms. Cumulatively, the need for a rehabilitation intervention spanning primary health care to independent activities of daily living is evident. Therefore, by definition, the support provided to survivors must be multifaceted, multidisciplinary, integrated, trauma- and violence-informed, and culturally safe.

Following BI, the recovery trajectory is strongly affected by the severity of the injury. In mild cases, 80% of individuals with a concussion or mild BI make a complete recovery. However, a portion of individuals experience persistent symptoms, cognitive impairments (eg, deficits in executive function and working memory, reduced processing speed and attention, difficulty in focusing, and difficulty with word recall), and reduced QoL for months to years after the injury. We propose that this gap in recovery is related to higher-order cognitive functions and, by targeting these core functions through rehabilitation and regaining function through the CSN rehabilitation intervention, individuals can return to a complete and meaningful life after the injury.

With all this in mind, this study protocol aims to evaluate a community-based, neurorehabilitative intervention to improve outcomes for survivors of IPV and IPV-BI.

### Objectives

The *primary objectives* of this study protocol are as follows:

1. To measure the efficacy of the CSN rehabilitative intervention in terms of its impact on resiliency, QoL, and neurocognitive functions
2. To evaluate the feasibility of the CSN rehabilitative intervention in terms of study recruitment, adherence, and retention

The *secondary objectives* of this study protocol are as follows:

1. To characterize neurocognitive function in women who have experienced IPV-BI
2. To characterize mental health consequences in women who have experienced IPV-BI

## Methods

### Design

This study will follow a pre- and postexperimental study design. Subsequently, a comparative study will be conducted by recruiting a control group receiving usual care.

### Participant Inclusion and Exclusion Criteria

Self-identified women (aged 18 to 50 years) who are survivors of IPV (disclosed) and IPV-BI (Brain Injury Severity Assessment [BISA] score ≥1) [5] will be eligible for recruitment to this study. Although IPV is not exclusive to women, this protocol study will only examine women. This approach was selected for two primary reasons: (1) the prevalence of reported IPV is higher in women compared to men [1] and (2) the infrastructure currently available for women who experience IPV is adequate to support recruitment into the intervention. The realities of recruiting self-identified women for a study of this nature are both impactful, from a survivor standpoint, and achievable, from a CSN approach.

Exclusion criteria will include current pregnancy; any diagnosed neurological disorder known to affect cerebrovascular, neurocognitive, or sensorimotor function (eg, stroke, Parkinson disease, Alzheimer disease, cerebral palsy, multiple sclerosis, migraine, or seizures); and taking medication that might alter blood pressure. Participants will be tested in the early follicular phase or, if they are on combined hormonal contraception, at the end of the week they have stopped this contraception.

### Recruitment

Recruitment will occur through partnerships with the Nanaimo Brain Injury Society (Nanaimo, British Columbia), the Central Okanagan Elizabeth Fry Society (Kelowna, British Columbia), William and Associates Counselling Services (Kelowna, British Columbia), and Connect Counselling and Therapy (Kelowna, British Columbia). Potential participants will be made aware of the project through promotional media and by direct staff referral within partner organizations. Potential participants will be screened by the research coordinator for eligibility using the adapted HELPS BI assessment tool [22].

### Ethical Considerations

The study has been reviewed and approved (H20-02412) by the behavioral ethics board at the University of British Columbia, Okanagan.

Participants who agree to take part in the study will be asked to provide informed consent and will be reminded that they can withdraw from the study at any time.

The identity of participants will be kept secure by storing consent forms and any other documents that could be used to determine the participants in a locked filing cabinet in the office of the senior author (PvD). Data will be deidentified and anonymized using codes so that participant information will remain confidential.

Participants will be compensated a total of CAD $550 (US $405.45) across the different components of the project for contributing their time and effort.

### CSN Rehabilitation Intervention

The CSN intervention is an evidence-based, multipillar, interdisciplinary rehabilitation program grounded in the ABI Wellness Brain Enhance And Recovery System (BEARS) program platform, with additional components incorporated to support the unique needs of survivors of IPV-BI.

#### The BEARS Program

##### Overview

The BEARS program [23] is a highly structured, noninvasive multidisciplinary rehabilitation program focused on improving cognitive capacity across a wide range of higher-order cognitive functions, resulting in a generalized improvement of cognitive ability and QoL. The BEARS program is designed based on the principles of neuroplasticity. In addition to the 10 general principles of neuroplasticity [24], the BEARS program includes the following five key components that promote neuroplastic changes: (1) *attention* (focused attention on the demands of a specific task), (2) effortful processing (task that is challenging enough that it requires effort to accomplish but not so difficult as to be discouraging), (3) *novelty/complexity* (task involving new learning with an appropriate level of complexity), (4) *differentiated stimulation* (specific tasks targeting a cognitive function), and (5) *sustained engagement* (sufficient time spent working on a particular cognitive task). The BEARS platform consists of 4 pillars (Textbox 1) that have been shown to improve function following BI from various causes [25-29].

**Pillars of the Brain Enhance And Recovery System (BEARS) platform.**

**Pillars**

- Aerobic exercise
  The BEARS aerobic exercise pillar aims to take advantage of the neuroplastic benefit of aerobic exercise to complement and accelerate the changes occurring as a result of cognitive training [25,29]. The aerobic exercise can include, but not be limited to, running, jogging, walking, rope jumping, swimming, stationary or recumbent bicycling, Zumba or dancing, using a treadmill or rowing machine, or elliptical exercises. The key factor for aerobic exercise is the release of neurochemicals that promote neuroplasticity and cognitive recovery. For this to be accomplished, it is critical that a specific exercise intensity is reached and maintained. The modality of the exercise is not important as long as the intensity level is met and maintained for a sufficient amount of time. A participant should be working at approximately 70% to 80% of their maximum heart rate for 30 minutes, or a minimum intensity of 12 on the Borg scale must be reached and maintained for a minimum of 30 minutes. At times, when participants are not able to exercise at this intensity or for the complete duration, the facilitator will work with the participants on improving their fitness level, aiming to enhance their intensity as high as possible. The aerobic exercise pillar will be monitored and recorded using an app embedded in the BEARS program. Facilitators will ensure that clients have been allowed to participate in physical activity by their physician or that the aerobic exercise is completed under the supervision of a certified professional (eg, physiotherapists and occupational therapists). ABI Wellness acknowledges that not all clients will be able to participate in the physical activity pillar; however, all possible accommodations will be made to allow for participation.
- Cognitive rehabilitation
  The cognitive rehabilitation pillar is focused on recovering and rebuilding higher-order cognitive functions, such as working memory, attention, and executive functions [23]. The cognitive training program used as part of the BEARS program is the Brainex program, which is a multiexercise program that specifically targets multiple brain regions and functions. The cognitive exercise being implemented is the Brainex symbol relations task, which is a computer-based cognitive exercise consisting of a sustained visual-spatial processing or relational reasoning task of progressively increasing difficulty. It requires participants to use relational reasoning on an analog clock to conceptually and automatically process relationships that increase in complexity. The participant enters the value, and feedback is presented. Once a criterion of 90% accuracy is attained over a series of consecutive responses, the complexity of the task increases. This cognitive exercise is not language dependent, so it is accessible to participants with different language backgrounds. Individually tailored goals will be set or adjusted for improving accuracy, increasing the number of correct responses per day, improving the number of sets completed per day, and lowering the time per set while maintaining accuracy. The best approach for optimal recovery adopted in this program is to focus on capacity-building rehabilitation and help individuals regain as much cognitive function as possible before using compensatory strategies to supplement and support continuing areas of impairment. Furthermore, the symbol relations assessment is incorporated within the program to measure the cognitive capacity of participants. A pilot intervention showed cognitive improvement in participants who had sustained a BI [26].
- Mindfulness meditation
  Regular mindfulness meditation has been shown to have a myriad of benefits to both the body and the mind. In particular, following BI, mindfulness can help with increasing self-awareness, and breathing techniques are very beneficial for helping to lower stress when feeling overwhelmed or frustrated [27]. Mindfulness meditation requires the client to participate in a guided meditation block that focuses on educating the client on various types of meditation and to develop their own familiarity and skill with meditation to begin practicing on their own. The critical factors for the mindfulness meditation exercise are regulation of attention, self-awareness, and engagement. The mindfulness meditation activity is monitored and tracked using the embedded app.
- Integrated health
  The BEARS program was developed to improve the quality of life (QoL) of individuals recovering from BI. The integrated health pillar is focused on bringing the behavioral, social, and emotional aspects of recovery and function together to complete the holistic and integrated recovery of the client. The integrated health pillar is composed of the (1) Traumatic BI (TBI)-QoL measurement system; (2) specific, measurable, attainable/achievable, realistic, and time-based (SMART) goal setting; and (3) transition planning. Using the TBI-QoL measurement system, the facilitator and the support team will be able to identify areas of concern that should be monitored or addressed during the program. The TBI-QoL measurement system is fully embedded into the BEARS program and is included as part of the intake process as well as the subsequent progress assessments. Data will be collected on each client throughout their time in the program. This allows for a deep level of understanding of the client’s initial level of function and the progress made throughout the program. A key factor of this program is effortful processing, meaning the more effort a client puts into the activities, the greater the improvement to be expected. In addition, QoL will be measured regularly to track progress in the domains of physical, emotional, cognitive, and social health. The TBI-QoL measurement system is accessible through the facilitator tracking system and the client portal. SMART goal setting allows the facilitator to help the client take small, manageable steps toward their various goals during their time in the program and teach them how to set goals for success. By working with the client with SMART goal setting, the facilitator will be providing the client with the necessary skills required to succeed upon completion of the program. In addition, transition planning is a critical component of all successful rehabilitation programs as this prepares the client for reintegration into the community and their future success.

##### Three Critical Factors of the BEARS Program

The 3 critical factors of the BEARS program are as follows:

1. *Setting goals:* The BEARS program creates standard goals for each pillar and exercise that act as reference points for ideal daily engagement. At times, when the standard goal may not accurately reflect an individual client’s ability or there may be limitations that prevent a client from reaching the standard goal, personal goals may be used to more accurately reflect the client’s engagement in the program.
2. *BEARS tracking system:* The BEARS program will be delivered through web-based applications available on Android tablets. The BEARS Facilitator app has been developed to fully integrate the 4-pillar approach and allow effortless client tracking and real-time data analysis for the facilitator. The app can be used for *organization, progress and engagement tracking, goal setting, program modification, and exercise settings*. The client portal will be used by clients to access the computer-based exercises. All client work done in the client portal will be automatically recorded, and data will be presented on the app. All administration functions of the BEARS program will be done through the BEARS tracking system.
3. *Client engagement:* Engagement is key in the BEARS program, and therefore, if a client is not fully engaged in a portion of the program, it is vital that the facilitator steps in and makes the necessary adjustments. At all times, the client’s progress, engagement, and goals will be monitored and adjusted based on their performance. The app will record and display client progress and engagement for every exercise within the program. It will provide an overview of weekly progress (against the benchmark expectations) and daily engagement goal (regarding the personal goal) graphs.

#### Additional Components

The purpose of integrating additional components into the BEARS program was to develop an IPV-BI–specific rehabilitation intervention for improving survivors’ cognitive function, brain health, and QoL. The additional components are described in Textbox 2.

**The additional components into the community support network (CSN) rehabilitation intervention.**

**Components**

- Trauma-informed approach or practice
  It is essential to integrate a trauma-informed approach (TIA) into the design and implementation of programs for survivors of intimate partner violence (IPV). Therefore, the BEARS program will be adapted for the community support network to be trauma informed. Integration of TIA addresses traumatic experiences with the goal of reducing symptoms, minimizing barriers to accessing health care, optimizing therapeutic outcomes, and working with survivors of IPV toward recovery. The four key elements of TIA (4Rs) are to (1) realize the trauma, (2) recognize signs of trauma, (3) respond through trauma principles, and (4) resist retraumatization. These are applied through six principles: (1) safety; (2) trustworthiness and transparency; (3) peer support; (4) collaboration and mutuality; (5) empowerment, voice, and choice; and (6) cultural, historical, and gender issues [30]. All staff who participate in and deliver the intervention will undertake specific and intentional orientation to TIA and language, particularly as they pertain to IPV. Furthermore, TIA will be achieved through the inclusion of a participant navigator who has lived experience with brain injury (BI) and career expertise working in the gender-based violence sector supporting survivors of IPV.
- Counseling
  The CSN will integrate a counseling component because survivors of IPV-caused BI often struggle with mental health issues. A great benefit has been observed with the inclusion of counseling into the rehabilitation program [31]. Therefore, targeted counseling of 60 minutes biweekly will be provided by clinical counselors who are trained in handling survivors of IPV and have extensive experience working with survivors of IPV.
- Inclusion of a navigator
  Because women who have experienced IPV are often dealing with a host of extra challenges, it is important that they have someone to connect with in addition to the BI experts delivering the rehabilitation program itself. The participant navigator will provide trauma- and violence-informed advocacy and program or systems navigation support to the participants as well as referrals to other community agencies or services as needed. The participant navigator will connect and support by phone or video call. Furthermore, the navigator will guide participants to maintain an activity logbook for 3 months after the completion of the intervention.
- Peer support
  The participant navigator will facilitate a monthly peer support group discussion, which will include personal development presentations from a variety of subject matter experts on topics, including navigating life after BI, healthy nutrition and lifestyle, art therapy, and the power of laughter in improving mental wellness.

### Measures

#### Demographics

Demographic information, including age, education level, race or ethnicity, socioeconomic status, gender, and sexual orientation will be obtained via self-report to accurately characterize participants.

#### BISA Interview

The BISA is a semistructured interview tool containing a series of questions regarding loss of consciousness and alterations in consciousness (eg, dizziness, memory loss, feeling stunned or disoriented, or seeing stars or spots) following potential BI due to IPV and provides a BI load summary score with a range of 0 to 8 (Table 1) composed of frequency, recency, and severity subscores [5,32]. The frequency score provides an estimate of the number of previous BIs, the recency score probes the time since the most recent event resulting in a BI and the time of the interview, and the severity score indicates whether a moderate-to-severe BI was ever sustained. The BISA tool has been useful in capturing the effects of IPV-BI on resting-state brain activation [32], white matter diffusions [13], brain morphology [33], cognitive-motor function [34], and BI symptoms [35]. Thus, the literature suggests that it will be appropriate to assess for previous BI and to assess a reliable subjective measure to allow strong inferences about BI and function.

**Table 1:** Scoring Brain Injury Severity Assessment (BISA) interview. Frequency (scale range 0-4), recency (scale range 0-3), and severity (scale range 0-1) are 3 subscales of the BISA tool. The BISA tool provides a summary score ranging from 0 to 8.

Subscales and score			Description
**Frequency**			
	0	There have been no previously reported brain injuries	
	1	There have been 1 to 5 previously reported brain injuries	
	2	There have been 6 to 10 previously reported brain injuries	
	3	There have been 11 to 15 previously reported brain injuries	
	4	There have been ≥16 previously reported brain injuries	
**Recency**			
	0	The event occurred >52 weeks ago	
	1	The event occurred between 27 and 52 weeks ago	
	2	The event occurred between 14 and 26 weeks ago	
	3	The event occurred within the past 13 weeks	
**Severity**			
	0	The presence of exclusively mild brain injuries	
	1	At least 1 episode involving loss of consciousness for >30 minutes or a period of posttraumatic amnesia for >24 hours had occurred (indicating moderate-to-severe brain injury)	

#### Mental Health Screening Tools

Each participant will undergo a mental health screen that includes clinical evaluations of abuse history, anxiety, depression, PTSD, substance use, and childhood trauma. These instruments listed in Textbox 3 will be explained and administered to each participant by the research coordinator.

**Mental health screening tools.**

**Tools**

- *Adverse Childhood Experience Questionnaire:* This is a 10-item questionnaire designed for administration to people aged ≥18 years. Questions cover family dysfunction; physical, sexual, and emotional abuse and neglect by parents or caregivers; peer violence; witnessing community violence; and exposure to collective violence [36]. It is a reliable [37] and valid [38] tool for the measurement of childhood trauma and negative health outcomes in adulthood. This was effectively used in exploring whether individuals exposed to physical and sexual abuse in childhood or domestic violence–prone environments are more likely to be involved in intimate partner violence (IPV) later in their life [39].
- *Conflict Tactics Scale-2 (CTS-2):* The CTS-2 (an updated version of the CTS tool) is a self-report 39-item survey measuring both the extent to which partners in a dating, cohabiting, or marital relationship engage in psychological and physical attacks on each other and their use of reasoning or negotiation to deal with conflicts [40]. Scoring for the CTS-2 involves assigning numerical values to responses and calculating subscale sores. Each question on the CTS-2 asks the participant to report the frequency of a specific behavior in the past year, with responses including 0=this has never happened, 1=once in the past year, 2=twice in the past year, 3=three to 5 times in the past year, 4=six to 10 times in the past year, 5=eleven to 20 times in the past year, 6=>20 times in the past year, and 7=not in the past year but it did happen before. To calculate a subscale score, the scores for each item in that subscale are added together. The CTS-2 has been shown to be valid and demonstrates reliable stability across its subscales: psychological aggression (*r*=0.69), physical assault (*r*=0.76), injury (*r*=0.70), and negotiation (*r*=0.60). However, the reliability coefficient for sexual coercion is noticeably weaker (*r*=0.30) [41].
- *Posttraumatic stress disorder (PTSD) checklist for Diagnostic and Statistical Manual of Mental Disorders (DSM)-5 (PCL-5):* The PCL-5 is a 20-item self-report measure that assesses the presence and severity of PTSD symptoms [42]. Items on the PCL-5 correspond with DSM-5 criteria for PTSD. The PCL-5 can be used to quantify and monitor symptoms over time, to screen individuals for PTSD, and to assist in making a provisional or temporary diagnosis of PTSD. The PCL-5 is composed of 20 items, each from 0 (not at all) to 4 (extremely). The total score for PCL-5 is calculated by adding together the scores for each of the 20 items. Each item is scored between 0 and 4, so the total score will range from 0 to 80. A cut-off score of 33 is often used, suggesting a high likelihood of PTSD [43]. The PCL-5 exhibits robust reliability, with impressive internal consistency (Cronbach α=0.94) and notable test-retest reliability (*r*=0.82) [42] and validity [44]. Furthermore, there was a strong relationship between current PTSD symptoms and meeting criteria for all 4 DSM-5 PTSD symptom clusters in women with IPV-caused brain injury [45].
- *Generalized Anxiety Disorder Scale (GAD-7):* The GAD-7 is a 7-item self-report inventory used for measuring the severity of anxiety [46]. The questions ask about common symptoms of anxiety the participant had during the past 2 weeks, such as worrying, being restless, being easily annoyed, and fearing. Each question is rated on a scale of 0 to 3, with higher scores indicating higher levels of anxiety. The total score ranges from 0 to 21. The GAD-7 is a quick and reliable tool for identifying the presence and severity of generalized anxiety disorder symptoms [46].
- *Patient Health Questionnaire-9 (PHQ-9):* The PHQ-9 is a 9-item self-report measure used to assess the levels of depression. The questions ask about common symptoms of depression the participant had during the past 2 weeks, such as feeling hopeless, having little energy, and difficulty concentrating [47]. Response options range from 0 (not at all) to 3 (nearly every day). The total score for the PHQ-9 ranges from 0 to 27, with higher scores indicating greater severity of depressive symptoms [48]. The validity and reliability of the PHQ-9 have been established in various studies [48]. The PHQ-9 questionnaire has been used to evaluate depression symptoms in individuals who have experienced IPV [49].
- *Alcohol Use Disorders Identification Test (AUDIT):* The AUDIT is a 10-item screening tool developed by the World Health Organization [50] to assess alcohol consumption, drinking behaviors, and alcohol-related problems. Each item in this tool is evaluated on a 0 to 4 scale, and the total score can range from 0 to 40 [51]. It is a valid and reliable tool for measuring alcohol-related problems [52].
- *Somatic Symptom Severity Scale (PHQ-15):* The PHQ-15 is a 15-item questionnaire that assesses somatic symptoms of pain across different body systems [53,54]. The tool uses a rating scale ranging from 0 (not at all) to 2 (nearly every day), and total scores range from 0 to 30, with higher scores indicating more severe somatic symptoms [54,55]. The PHQ-15 is a reliable tool with a test-retest reliability of *r*=0.60 [56]. It has also been used to show that survivors of IPV have greater levels of somatic symptoms [57].
- *International Trauma Questionnaire (ITQ):* The ITQ is an 18-item questionnaire that assesses PTSD and complex PTSD [58]. The reliability of the ITQ has been well established, as evidenced by several publications [59,60]. Furthermore, it has been used in studies to assess the relationship between complex PTSD and the severity of IPV psychological perpetration [61,62].
- *Brief Irritability Test (BITe):* The BITe is a 5-item questionnaire assessing common feelings of irritability [63]. The BITe consists of questions regarding how the individual has felt in the past couple of weeks, and they rate their experience on a scale from 1 (not at all) to 6 (always). The score ranges from 5 to 30, with higher scores indicating higher levels of irritability.
- *Level 2-Substance Use-Adult:* This questionnaire is a 10-item screening tool developed by the US National Institute on Drug Abuse to assess substance use [64]. The participant rates each item on a 5-point scale, from 0=never to 4=nearly every day. Scores on the individual items are interpreted independently because each item inquires about the use of a distinct substance. The rating of multiple items at scores >0 indicates greater severity and complexity of substance use.

#### Outcome Measures

The following outcome measures, which will be administered by a trained member of the research team, will be used to assess resiliency, QoL, and neurocognitive function:

1. *Resiliency:* Resiliency will be characterized using the Connor-Davidson Resilience Scale [65]. This 25-item questionnaire measures responses to 5 factors associated with resilience on a 5-point Likert scale ranging from 0 (not true at all) to 4 (true nearly all the time). The scale has been shown to have good psychometric properties, with high internal consistency and test-retest reliability [65].
2. *QoL:* QoL will be measured using a modified version of the Medical Outcomes Study short form-36 [66]. This 36-item questionnaire consists of 8-scaled scores covering several aspects of QoL and general health. Each scale is scored separately and transformed onto a scale that ranges from 0 to 100 for ease of comparison, with lower scores indicating a lower QoL.
3. *Neurocognition:* Neurocognitive function will be assessed using the Creyos (formerly known as Cambridge Brain Sciences) tool [67], which consists of 12 different tasks that assess aspects of cognition, including reasoning, memory, attention, and verbal ability. The tasks include digit span, double trouble, feature match, grammatical reasoning, monkey ladder, odd one out, paired associates, polygons, rotations, spatial planning, spatial span, and token search. The tasks have been validated in studies of patients, in brain imaging studies of healthy volunteers, and in several large-scale public studies [67-69]. The Creyos tool maintains a global normative database of >75,000 participants that allows for detailed comparisons of individuals to specific populations. The tests have been shown to be valid and reliable, and a meaningful change can be calculated using the normative database [67]. Notably, all tasks, which users report to be fun and engaging, will be administered through web application or in person using a desktop or laptop and require no expert technical support to administer. Task results will be stored securely in the cloud and can be easily downloaded for offline analyses [65,69].

In addition to the tasks included in the Creyos tool, a task-switching paradigm will be used to probe executive functions [70]. A simplified version of the task that is run on an Android tablet will be used to capture executive dysfunction in this study. This simplified version has been implemented in a study (unpublished) examining head impacts and concussions in ice hockey players.

Furthermore, we will have access to the following measures collected throughout the intervention, which are incorporated within the BEARS program [23]:

1. *Traumatic BI-QoL (TBI-QoL) measurement system:* The TBI-QoL measurement system is a recently developed QoL measure specifically designed for people who have experienced TBI. It consists of a 20-item questionnaire covering the domains of physical health, emotional health, cognitive health, and social participation [71]. The findings of this tool would be helpful in corroborating the findings of the study short form-36.
2. *Brainex cognitive assessment:* This is a series of tests of neurocognitive function, including assessments of symbolic thinking. The findings of this tool will be helpful in strengthening the findings of the Creyos assessment.
3. *Functional performance indicators:* The functional performance indicators are independently derived by ABI Wellness and act as additional composite scores that are purely indicative and aim to reflect the ability of the individual to function in everyday life. The scores are created from various TBI-QoL subdomains, which were chosen to best reflect these functional performance measures.
4. *ABI Wellness overall engagement:* The overall engagement assessment is done to provide an overview of the weekly and cumulative engagement across the different activities of the ABI Wellness 4-pillar program. The engagement is measured against the personal goal of each activity set by the facilitator.

#### Feasibility Measures

Study recruitment, adherence, and retention will be used to assess feasibility. Recruitment will be measured as the proportion of individuals enrolled compared to those who were approached and eligible but did not enroll. The proportion of weekly sessions attended by the participants will be calculated to assess adherence. Retention will be evaluated by the proportion of participants with complete data sets on primary outcome measures at pre and postintervention time points. Withdrawing will be calculated at the postintervention time point as the proportion of the participants who will withdraw from the intervention [72]. In addition, we will be collecting qualitative feedback and anecdotal information on how participants like the program, what they think about the program overall, and which component of the intervention was most or least enjoyed.

### Procedure

#### Overview

**Figure 1:**
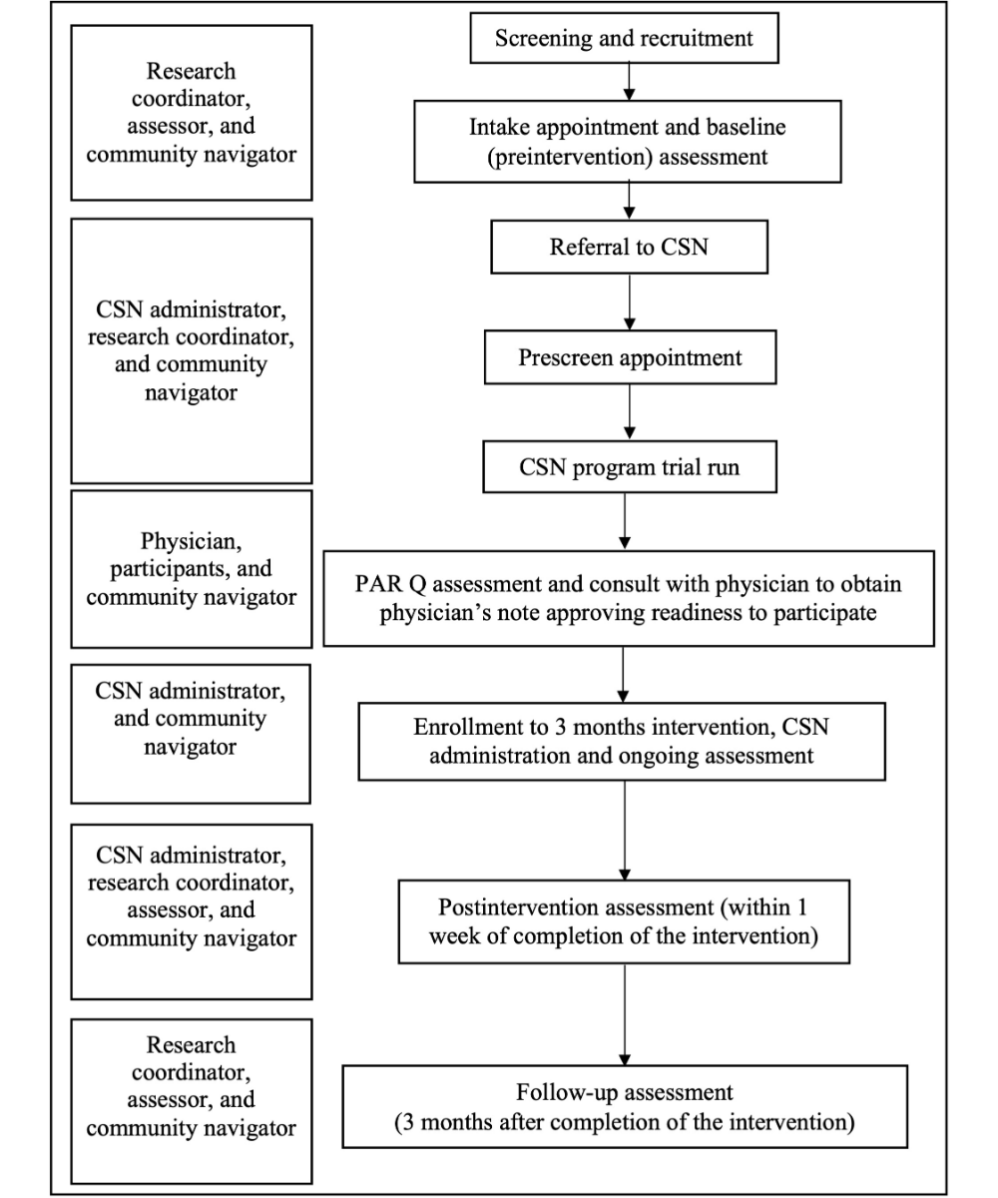
Steps to participation, enrollment, and assessment with the program team. Right side of the figure provides various steps of the study, and the left side shows roles of the study team at various stages. CSN: community support network, PAR-Q: Physical Activity Readiness Questionnaire.

The intervention program and assessments will be in person or remotely operated (on a university-licensed Zoom app), or a hybrid combination of in person and remote operation based on participants’ preference, their location, and context such as public health orders. After the research coordinator explains what is involved in the study, if a client is willing and eligible to participate, informed consent will be obtained. All participants will undergo 4 testing sessions: the first and second sessions will occur before enrollment in the intervention, and the third and fourth sessions will occur within 1 week and 3 months of the end of the intervention, respectively. In the first session (lasting approximately 2.5 hours), participants will complete a mental health screening battery. In the second, third, and fourth sessions (lasting approximately 1.5 hours), they will complete tests of resiliency, QoL, and neurocognition. If, as a result of the assessments, a clinically significant abnormal finding is obtained, the participant will be directed to seek medical advice from a health care provider. Various steps of the study and the roles of the study team are outlined in Figure 1.

#### Structure of the CSN Intervention

The dose and sequence of the CSN rehabilitation intervention is outlined in Table 2.

**Table 2:** An example of a 2 days per week schedule of the community support network rehabilitation intervention. The table provides the administration sequence and duration of aerobic exercise, cognitive training, and mindfulness meditation with an additional note on monthly peer group support discussion and biweekly counseling sessions.

Time	Duration^a^ (total duration/day: 2.5 hours)	Activities
9:30 to 10 AM	30 minutes	Aerobic exercise
10 to 10:10 AM	10 minutes	Break
10:10 to 10:50 AM	40 minutes	Cognitive training, block 1
10:50 to 11:10 AM	20 minutes	Mindfulness meditation
11:10 to 11:50 AM	40 minutes	Cognitive training, block 2
11:50 AM to noon	10 minutes	Wrap up

^a^Participants must commit to attending the program on a part-time basis for 3 months. The program is adaptable, with an example 2 days per week version outlined in this table.

The following activities will be provided to the participants in addition to the activities mentioned in Table 2:

- There will be a peer group support group session for approximately 60 minutes once a month.
- The program includes counseling of approximately 60 minutes twice a week.
- There will be a participant navigator who provides trauma- and violence-informed advocacy and program or systems navigation support to participants and referrals to other community agencies or services as needed.

#### Program Team

All members of the program team will receive foundational training in IPV and trauma-informed practice. The CSN facilitators will take formal training on the BEARS program from ABI Wellness and will be certified for its administration. The research coordinator will receive formal training in the administration of the various assessment tools. The team will meet every other week, or more frequently as needed, to track the program and participant progress and address questions or issues that arise.

### Sample Size Estimation

To generate and design a study with adequate power, we estimated the required sample size based on previous data from our laboratory in both healthy participants and those with IPV-BI [34,35]. Power calculations are based on the desired end point to detect a change in each of the assessments across testing times. Therefore, assuming our published SDs for these measures and considering the potential for increased variability in this study population, a total sample size of 100 participants would provide a >90% probability that the study will detect a significant change score at a 2-sided 5% significance level.

### Statistical Analysis

The objective of this project is to evaluate the efficacy of a CSN intervention for women who have experienced IPV-BI. This will be accomplished by quantitatively measuring resiliency, QoL, and neurocognition before and immediately after the intervention. A follow-up assessment will occur 3 months after the completion of the intervention to evaluate the maintenance of any gains in function. The mean (or median) for each assessment will be compared across testing times using 1-way ANOVAs. Relationships between BISA scores and the change scores for each assessment will be evaluated using regression analysis. Furthermore, the outcome of the intervention will be compared among various subgroups based on the findings of various mental health screening tools (eg, participants with PTSD vs participants without PTSD), substance use, and adverse childhood experience (eg, participants with adverse childhood experience vs participants without such experience). Various demographics and mental health screening assessments will be used to control for nonspecific effects of IPV. In addition, we will have opportunities to better characterize IPV-BI based on the BI load from the BISA tool, findings of various mental health screening tools, and cognitive findings using the Creyos assessment and task-switching paradigm. The α value will be set a priori at *P*<.05. All data will be analyzed using SPSS (IBM Corp).

### Potential Risks, Safety, and Security

We will take appropriate precautions to minimize potential risks associated with the proposed study. The participants are not expected to encounter any substantial physical or mental risks as a result of this study. The BISA and other psychosocial assessments will be conducted by a trained research coordinator who has substantial career experience directly supporting survivors of IPV, previously at a women’s shelter and currently at a community agency as a victim support worker. She is well-versed in trauma- and violence-informed practice and will take all the necessary steps to protect participant safety and well-being. In addition, the assessments will be conducted at the community agency with which the participants are already familiar and where trauma support services are in place and readily available should they be required. We will also develop a series of participant safety protocols that outline how the research coordinator will identify suicidal ideation and risk factors for suicide, participant safety, and child safety and what actions she will take if required. All participants will be undergoing clinical counseling as part of the study and may have received professional support resulting from their trauma and abuse. Nonetheless, completing the testing involved in this study may trigger painful memories and create a triggering state. If the participant expresses any concerns during the data collection that suggest the need for immediate attention, they will be counseled by the research coordinator or participant navigator, both of whom will have strong professional backgrounds supporting survivors of gender-based violence. The assessment and intervention administration will be based on the trauma-informed approach (TIA). Research team members will be trained to follow TIA. All interviews and mental health screening instruments will be completed with each participant privately. The mental health screening process will be conducted by the research coordinator who will have to complete specific training and practice with all instruments and interview schedules.

## Results

This study is in the testing phase. As of October 2023, we enrolled 6 participants. We anticipate that the CSN rehabilitation intervention will be effective in improving resiliency, QoL, and neurocognitive function in women who have experienced IPV-BI. Furthermore, we also anticipate that this intervention will be feasible in terms of study recruitment, adherence, and retention. This study will also be able to characterize, more generally, neurocognitive and psychopathological consequences in women who have experienced IPV-BI. We will report the results in follow-up papers.

## Discussion

### Principal Findings

To the best of our knowledge, this will be the first study to systematically investigate the efficacy of an evidence-based, multipillar, interdisciplinary rehabilitation program in improving resiliency, QoL, and neurocognitive functions in women who have experienced IPV-BI. All components of the CSN program are evidence based and have shown a positive impact on QoL in people with TBI and those with various other forms of BIs [26-29,31]. This intervention also aims to take advantage of the neuroplastic benefits of its various components [24,25,29]. In addition, this intervention will be administered based on TIA that addresses traumatic experiences with the goal of reducing symptoms and optimizing therapeutic outcomes [30]. Therefore, we anticipate improvement in resiliency, QoL, and neurocognitive functions with the CSN intervention in women who have experienced IPV-BI. Moreover, we expect that the CSN intervention will be feasible in terms of recruitment, adherence, and retention because it has been successfully implemented to address physical and cognitive impairments in individuals with BI from various causes. Furthermore, the CSN is IPV-BI specific in that it is structured with the addition of TIA, counseling, a participant navigator, and peer support.

This efficacy and feasibility study will help to address the current gap in the intervention study aimed at improving resiliency, QoL, and neurocognitive functions in women who have experienced IPV-BI. Successful implementation will help to support future full-scale comparative studies and randomized controlled trials.

### Limitations

The proposed study will not be without limitations. The lack of a comparative control group will be a limitation. Having a comparable control group to an IPV-BI intervention group is a substantial challenge. Furthermore, an IPV-BI–specific gold standard rehabilitation intervention is not available, which makes a comparative study with a control group even more challenging. However, the addition of an appropriate control group would obviously strengthen the outcome of the CSN intervention; therefore, it is a recommendation for future studies. Having limited recruitment sites within a specific geographical region could limit the pool of participants and the generalizability of the findings of this study.

### Conclusions

This will be the first intervention study of its kind to examine the impact of a rehabilitation intervention on resiliency, QoL, and neurocognitive function in women who have experienced IPV-BI. If this intervention is successfully implemented and its effectiveness is demonstrated, the findings will not only benefit individual participants and their families but could also have far-reaching implications for policy makers, service seekers, and service providers.

## Abbreviations

BEARS: Brain Enhance And Recovery System
BI: brain injury
BISA: Brain Injury Severity Assessment
CSN: community support network
IPV: intimate partner violence
IPV-BI: intimate partner violence–caused brain injury
NFS: nonfatal strangulation
PTSD: posttraumatic stress disorder
QoL: quality of life
TBI: traumatic brain injury
TIA: trauma-informed approach

## Data Availability

The data sets generated or analyzed during this study are not publicly available due to the sensitivity of the data but are available from the corresponding author on reasonable request.
